# Impact of multiple bird partners on the seed dispersal effectiveness of China’s relic trees

**DOI:** 10.1038/srep17489

**Published:** 2016-01-04

**Authors:** Ning Li, Xin-hai Li, Shu-qing An, Chang-hu Lu

**Affiliations:** 1 Laboratory of Plant-Animal Interactions, College of Biology and the Environment, Nanjing Forestry University, Nanjing 210037, China; 2 School of Life Science, Nanjing University, Nanjing 210046, Jiangsu, China; 3 Institute of Zoology, Chinese Academy of Sciences, Beijing 100101, China

## Abstract

Frugivorous birds generally exhibit an unequal contribution to dispersal effectiveness of plant species as a function of their habitat adaptation and body size. In our study, we compared the effectiveness of multiple bird species that contribute to the dispersal of the endangered relic Chinese yew, *Taxus chinensis*. Seven bird species dispersed *T. chinensis* seeds, with *Picus canus*, *Turdus hortulorum*, and *Urocissa erythrorhyncha* being the main dispersers. The quantity part of dispersal effectiveness was strongly influenced by two inherent characteristics of disperser species: body size and habitat adaptation. However, the quality part of dispersal effectiveness was only influenced by disperser type. For instance, small generalist birds and large specialist birds removed more seeds than other type dispersers. Moreover, small birds and specialist birds contributed slightly more to the dispersal quality of *T. chinensis* than large birds and generalist birds respectively; however, these differences were not significant. Our results suggest that dispersal effectiveness is affected by variety in the body size and habitat adaptation of different dispersers. Therefore, such variation should be incorporated into spatial and temporal management actions of relic plant species in patchy, human-disturbed habitats.

Human-disturbed landscapes dominate most terrestrial ecosystems globally^1^. In eastern Asia and Europe, human land-use intensification has been particularly strong, resulting in the continuous loss of natural habitats, leading to many isolated stands of tree species remaining in highly human-disturbed habitats^2^,^3^. Therefore, it is important to understand how such tree species interact with frugivorous birds, and how these interactions influence the regeneration fate and persistence of these trees in disturbed habitats^4^,^5^,^6^.

In general, many tree species in human-disturbed habitats are foraged on by multiple local bird species^7^, which may play an important role as seed dispersers for these species^8^,^9^,^10^. Usually, two components of dispersal effectiveness are used to evaluate the contribution of an effective disperser species that contributes to tree regeneration; specifically, quantity and quality of seed dispersal^11^. Quantity is determined by the number of visits by bird dispersers to the seed source and the number of seeds dispersed per visit. In comparison, quality is primarily determined by seed treatment during transport and the chance of seeds deposited in a suitable habitat^11^,^12^,^13^,^14^. Thus, most theoretical and empirical studies have demonstrated that different dispersers have different dispersal effectiveness, which is determined by two key inherent characteristics, body size and habitat adaptation^15^,^16^. Larger birds tend to exhibit better dispersal effectiveness than smaller birds, with habitat generalist birds being higher quality dispersers than habitat specialist birds^15^,^16^,^17^,^18^. However, increasing scientific evidence supports the concept that dispersal effectiveness of bird dispersers was strongly influenced by multiple inherent characteristics, including body size and habitat adaptation^13^,^14^. Due to the inherent difficulty of studying multiple dispersers, empirical evidence supporting the generality of the dispersal effectiveness of multiple dispersers is limited.

In our study, we quantified two characteristics of multiple disperser species (body size and habitat adaptation) in addition to their dispersal effectiveness (quantity and quality)^12^,^13^, in a natural frugivorous bird–plant system. We selected a subtropical forest ecosystem as a typical human-disturbed habitat, and focused on the seed dispersal of a fleshy-fruited relic tree species, the Chinese yew (*Taxus chinensis*), by four bird partners; the Chestnut bulbul *Hypsipetes castanonotus*, the Grey-backed thrush *Turdus hortulorum*, the Grey-faced woodpecker *Picus canus*, and the Red-billed blue magpie *Urocissa erythrorhyncha*. We hypothesized that multiple bird partners vary in their effectiveness because of differences in body size or habitat adaption respectively. In addition, we also tested whether these two inherent characteristics of disperser species influences their seed dispersal effectiveness.

## Results

### Diversity of frugivorous birds

During the fruiting season, we observed 20 habitat generalist species (*n* = 196 individuals) and 19 specialist species (*n* = 260 individuals) within the *T. chinensis* habitat in Tongkeng. Species diversity did not differ between the generalist and specialist assemblages; species richness and all three diversity and evenness indices were similar in both assemblages (Table 1; Table S1).

### Quantitative dispersal effectiveness by multiple bird dispersers

During the fruiting season, seven bird species (year: visits) (2011: 298; 2012: 307) were observed to disperse *T. chinensis* seeds. The main dispersers were the *P. canus*, *T. hortulorum*, and *U. erythrorhyncha*, with this information being determined from seed removal rates (Table 2).

**Figure 1:**
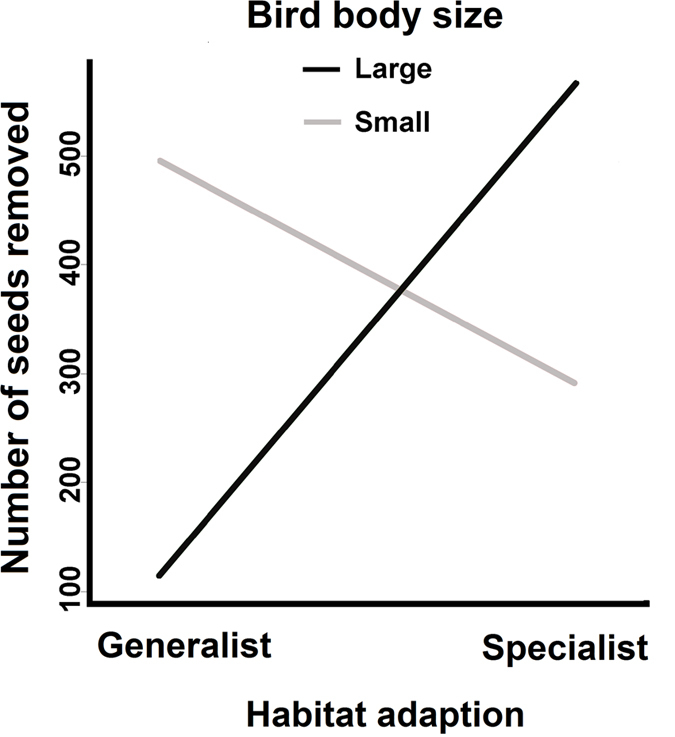
A generalized linear mixed-effects model showing the association of *Taxus chinensis* seed removal with dispersers’ body size and habitat adaptation in Tongkeng, east China.

The generalised linear mixed-effects model showed that the quantity of seed dispersal was strongly affected by the interaction term of body size and habitat adaptation by each disperser (*F* = 1035.54, *df* = 1, *P* < 0.0001). Specifically, small generalist birds and large specialist birds removed more seeds (Fig. 1). The interaction term explained 96.9% variance of the amount of removed seeds, whereas the linear terms of habitat adaptation and body size contributed minimally.

### Qualitative dispersal effectiveness by multiple bird dispersers

**Figure 2:**
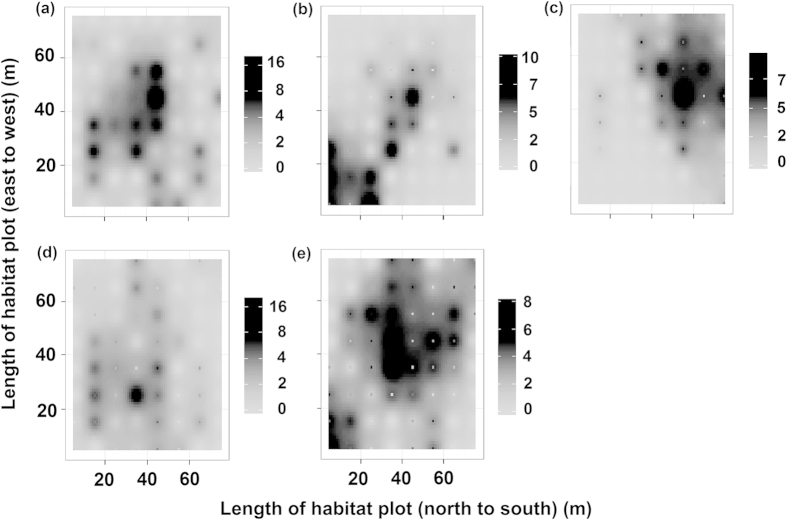
Distribution of 1-year-old seedlings of *Taxus chinensis* trees (a) and perching patterns of the four disperser species (b–e) in Tongkeng, east China. (**a**) Seedling number of Chinese yew *Taxus chinensis*; (**b**) Perching pattern of Grey-faced woodpecker *Picus canus*; (**c**) Perching pattern of Grey-backed thrush *Turdus hortulorum*; (**d**) Perching pattern of Red-billed blue magpie *Urocissa erythrorhyncha*; (**e**) Perching pattern of Chestnut bulbul *Hypsipetes castanonotus.* 245 seedlings for (**a**), and 251, 270, 412, 366 perching frequency of birds for (**b**–**e**) respectively. Coloured contours are interpolated from the value of the corresponding variable in the centroid of each habitat cell. The colour scales are shown.

The seedling census recorded 245 seedlings in Tongkeng Village (Fig. 2a). The seedling distribution pattern was influenced by the perching pattern of the bird dispersers (Fig. 2).

**Figure 3:**
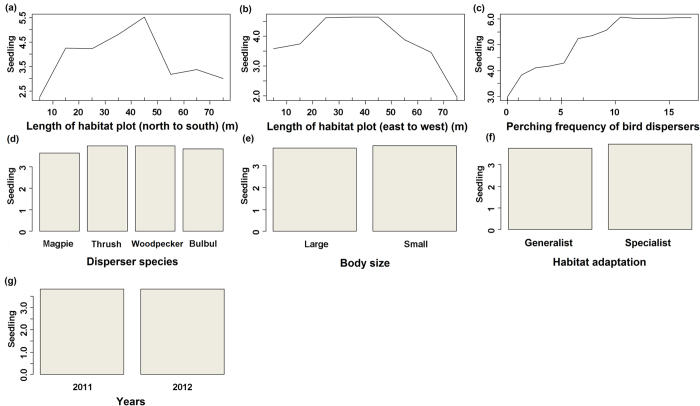
A machine learning algorithm, random forest, for testing the association of *Taxus chinensis* seedling numbers with habitat plot (a,b), perching frequency of dispersers (c), disperser species (d), dispersers’ body size (e), dispersers’ habitat adaptation (f), and surveying year (g) in Tongkeng, east China. It shows the partial effects of several independent variables on the number of seedlings, based on the random forest model. Bars show means. Disperser species: bulbul, Chestnut bulbul *Hypsipetes castanonotus*; magpie, Red-billed blue magpie *Urocissa erythrorhyncha*; thrush, Grey-backed thrush *Turdus hortulorum*; woodpecker, Grey-faced woodpecker *Picus canus.*

After foraging, four disperser species exhibited different perching patterns (years: behaviours), with 412 post-foraging behavioural observations for *U. erythrorhyncha* (2011:193; 2012: 219), 251 observations for *P. canus* (2011: 127; 2012: 124), 270 observations for *T. hortulorum* (2011: 140; 2012: 130), and 366 observations for *H. castanonotus* (2011: 195; 2012: 171) (Fig. 2b–e). The generalised linear mixed-effects model indicated that perching by the four dispersers significantly contributed to the number of documented seedlings (*F* = 315.53, *df* = 1, *P* = 0). The model also showed that the linear terms of disperser species (i.e. body size and habitat adaptation) and their interaction terms minimally contributed to the seedling numbers (habitat adaptation: *F* = 7.72, *df* = 1; body size: *F* = 10.27, *df* = 1; habitat adaptation: body size: *F* = 0.89, *df* = 1). The results of the random forest analysis showed a clear positive association between perching by birds and seedling numbers. The contributions of species, disperser body size, and disperser habitat adaption to dispersal quality were similar. In contrast, small birds and specialist birds contributed slightly more to the dispersal quality of *T. chinensis* than large birds and generalist birds; however, these differences were not significant (Fig. 3).

## Discussion

This study showed that *T. chinensis* trees that grow in human-disturbed habitats interact with a broad variety of bird species. We demonstrated that the quantitative components of dispersal effectiveness of multiple bird partners with a single plant varies as a function of their two inherent characteristics (body size and habitat adaptation), as well as with respect to the quality components as a function of their disperser type (representing a single inherent characteristic).

It is not surprising for *T. chinensis* trees to attract diverse bird species to forage and disperse its seeds in the human disturbed habitat, because the features of frugivorous bird species surrounding the focal trees clearly place them in the bird-yew system, as outlined by Li *et al.*^7^,^19^,^20^. For this reason, it is easy for many plants with fleshy fruits to develop effective dispersal interactions with resident fauna in disturbed habitats^6^,^9^,^21^.

Our results indicate that multiple bird partners exhibit complex foraging patterns based on their body size and habitat adaptation, with these characteristics resulting in different bird species contributing differently to dispersal quantity. As expected, small generalist birds and large specialist birds contributed the most to seed dispersal quality. This phenomenon is associated with the foraging behaviours of birds that inhabit disturbed habitats, supporting the results of previous studies^8^,^15^. For instance, small generalist birds often flock together to forage, which may decrease the predation risk in disturbed habitat. Moreover, large specialist birds tend to spend more time in trees to acquire more food^15^. These two foraging behaviours exhibited by birds in disturbed habitats may explain why these two types of dispersers contributed more to dispersal quantity than other types of birds.

Our results also suggested that bird perching behaviour has a strong influence on dispersal quality in disturbed habitats. Perching behaviour of the four most common dispersers was positively associated with the number of 1-year-old seedlings. This result is supported by previous studies showing that frugivorous birds use perches for vigilance, resting, and shelter, leading to perches receiving a disproportionate number of seeds after repeated use^22^,^23^,^24^. The four most common dispersers detected in the current study also exhibited complex post-foraging behavioural patterns, which were a function of body size and habitat adaptation, resulting in birds contributing differently to dispersal quality. For instance, perching habitat used by small birds and specialist birds was strongly associated with that of seedling habitat, resulting in their contribution to dispersal quality being higher than that of other bird types; however, this difference was not significant. This difference may be influenced by differences in habitat selection among bird species, leading to an unequal contribution to quality. This issue is highlighted by the hypothesis of “directed dispersal”^6^,^13^,^25^,^26^, whereby once a bird species shows a higher preference for perches in seedling suitable habitat, this bird species becomes a high-quality disperser species, because most seeds reach suitable habitat^6^,^13^. Moreover, observing the post-foraging behavior of the birds requires extensive effort. In our study, we chose the four most common birds as a priority, trying to obtain the whole information of seed removal by the four bird species, rather than part of the information for the entire community. Missing the other seed removers may cause bias, yet the bias should not be large.

Our results indicate that differences in the body size and habitat adaptation of disperser species are common, representing an important component for plant regeneration; thus, disperser characteristics should be explicitly considered in future studies of disperser effectiveness and in the management of relic plants in patchy habitats.

## Materials and methods

### Species and study site

The Chinese yew, *T. chinensis*, is an endemic gymnosperm species of China. This species is designated as “endangered” in the International Union for Conservation of Nature (IUCN) Red List (2013), and is ranked as a first priority protected species by the Chinese Government. The Chinese yew is widely distributed in eastern China. However, the wild *T. chinensis* population has a weak regeneration capacity, due to low pollination rates, seed-predator pressure, weak competitive ability of seedlings, and a scarcity of viable microhabitats for recruitment^27^.

The population of *T. chinensis* in Tongkeng village (30° 00′ N, 119° 22′ E; 553–638 m above sea level [a.s.l.]) is the largest natural population in Zhejiang Province, China^28^. The population contains 158 individuals, consisting of 68 female and 90 male trees. The vegetation surrounding the *T. chinensis* population is highly fragmented because of long-term habitat use by humans. Human-disturbed patches of fir (*Cunninghamia lanceolata*), bamboo (*Phyllostachys heterocycla*), and cypress (*Cupressus funebris*) are interlaced with Carya (*Carya cathayensis*), tea (*Camellia sinensis*), and the residential area to form a heterogeneous landscape surrounding *T. chinensis*^20^.

### Diversity of bird assemblages

To quantify the diversity of frugivorous birds within the *T. chinensis* habitat, we estimated the species richness, diversity, and total abundance of frugivorous birds during the fruiting season using line transects. One transect (50 m wide × 3 km long) was established through the *T. chinensis* population, and two parallel transects of the same dimensions were established 100 m on either side of the first transect. Two investigators walked along each transect between 07:00 and 10:00 and between 16:00 and 18:00, counting the number of frugivorous birds^19^. Each transect was investigated every three days. We retained the highest value of recorded individuals during six censuses for each frugivorous species. Habitat generalist species were classified as species that use multiple habitat patches such as mother tree source patch, bamboo patch, and fir patch in disturbed habitat, whereas specialist species primarily use a single habitat patch, usually within the source patch^16^,^20^. The Shannon–Wiener index, Pielou species evenness index, and Simpson’s dominance index were used to compare species diversity between generalist and specialist assemblages^29^,^30^.

### Quantitative dispersal effectiveness by multiple bird dispersers

Ten mother trees were monitored in two *T. chinensis* fruiting seasons (2011 and 2012)^19^. Each mother tree was observed for an 8-h period, starting at sunrise. Observations were made with binoculars from a hide placed at least 30 m from the trees, and ended when no more fruit remained on the mother trees. All observations were made under good weather conditions.

For each bird that visited a mother tree, we recorded the species, visiting frequency, foraging amount per visit, and the fruit-handling behaviour from the arrival to the departure of each bird. If a group of conspecific birds visited the tree and the behaviour of all birds could not be observed simultaneously, we focused on the individual that was most visible^31^,^32^. We defined generalists and specialists by their habitat adaptation, small birds and large birds by their weight^33^, and we qualified seed dispersers by their fruit-handling behaviour^34^,^35^. Then, we used a generalised linear mixed-effects model to test whether disperser body size, habitat adaptation, and their interaction term influenced dispersal quantity (the summed number of visits per species across the two years multiplied by the average number of fruit foraged per visit).

### Qualitative dispersal effectiveness by multiple bird dispersers

The quality of dispersal effectiveness among different disperser species was analysed. We were able to collect reliable data on the foraging and post-foraging behaviors of the two most common generalist dispersers (*U. erythrorhyncha*, mean weight: 180 g; *H. castanonotus*, mean weight: 40 g) and the two most common specialist dispersers (*P. canus*, mean weight: 130 g; *T. hortulorum*, mean weight: 50 g)^20^,^33^. The quality of seed dispersal effectiveness was evaluated by testing the spatial correlation between bird perching frequency and the quantity of 1-year-old seedlings. We examined this correlation because the perching frequency of the different dispersers indicates their ability to deposit seeds into suitable habitat for the yew, thus causing a differential contribution to 1-year-old seedlings, representing “effective dispersal”^19^,^24^.

A habitat plot of 80 m × 80 m was established according to the slope aspect of the study site, with focal mature trees occupying the centre. Habitat cells of 10 × 10 m^2^ were used to digitise the study site. One-year-old seedlings (H ≤ 10 cm) in each cell were then located. Overall, 64 sampling cells were surveyed at the study site. We observed the post-foraging behaviour of the four selected bird species, and recorded perching locations of each bird species in the 80 m × 80 m habitat plot after they left the mother trees. The perching locations of each bird were recorded every 30 s, until the bird was lost from sight^19^,^32^. These perching locations were used to identify characteristic landmarks near the mother trees for later verification of estimates through measurements on maps.

We used the function to perform univariate Kriging metamodels to interpolate seedling number and bird perching frequency in the sampled habitat plot. The method used for spatial interpolation is the krige function in R package gstat^36^. To test dispersal quality by multiple bird dispersers, we first used a machine-learning algorithm (random forest model) to plot the partial effect of bird perching frequency, disperser body size, disperser habitat adaptation, habitat plot, survey years, and seedling numbers (R package Random Forest)^37^. Then, the number of seedlings in the habitat cells was explained in a generalised mixed effect model, in which the perching frequency of the bird disperser, body size, and habitat adaptation were the covariates, while species, surveying year, habitat cells, and their interaction term were the random effects. We used the function glmer in the R Ver. 3.1.2 package ‘lme4’ for the analysis. Because the numbers of seedlings are count data, we selected the Poisson distribution in glmer.

## Additional Information

**How to cite this article**: Li, N. *et al.* Impact of multiple bird partners on the seed dispersal effectiveness of China’s relic trees. *Sci. Rep.* **6**, 17489; doi: 10.1038/srep17489 (2016).

## Supplementary Material

Supplementary Information

## Figures and Tables

**Table 1:** Diversity comparison between generalist and specialist assemblages within the *Taxus chinensis* habitat in Tongkeng, east China.

Measure of diversity	Generalistassemblage	Specialistassemblage
Shannon-Wiener diversity	2.76	2.64
Species evenness index	0.92	0.90
Simpson’s dominance index	0.07	0.09

**Table 2:** Comparison of quantitative dispersal effectiveness by multiple bird dispersers in Tongkeng, east China.

Bird species	Body size	No. visits		Fruits foraged per visit
		2011	2012	
Generalist birds				
* Urocissa erythrorhyncha*	Large	72	84	6.4 ± 1.9
* Hypsipetes castanonotus*	Small	37	30	5.0 ± 1.5
* Hypsipetes mcclellandii*	Small	19	11	3.3 ± 0.8
Specialist birds				
* Picus canus*	Large	78	75	9.4 ± 2.3
* Turdus hortulorum*	Small	62	70	8.7 ± 1.7
* Zoothera dauma*	Large	23	24	5.0 ± 1.0
* Myophonus caeruleus*	Large	7	12	2.8 ± 0.4

Seed dispersers are birds that swallow entire fruits, defecating or regurgitating the seeds^35^. Unit time is an 8-h period starting at sunrise. Results are presented as the means ± SE.
