# Supplementary material for: Impact of multiple bird partners on the seed dispersal effectiveness of China’s relic trees
Source: Sci Rep. 2016 Jan 4;6:17489. doi: 10.1038/srep17489 (PMC4698718; doi:10.1038/srep17489)
Supplement: Supplementary Information [file srep17489-s1.doc]

**Impact of multiple bird partners on the seed dispersal effectiveness of China’s relic trees**

Ning Li ^1^, ^2^, Xin-hai Li^3^, Shu-qing An^2^, Chang-hu Lu^1^

**Table S1** **Forest generalist and specialist observed within the habitat of *Taxus chinensis* in the fruiting season of 2011 and 2012**

| Habitat generalist | Individuals | Habitat specialist | Individiuals |
| --- | --- | --- | --- |
| *Anthus hodgsoni* | 13 | Abroscopus albogularis | 12 |
| *Anthus sylvanus* | 3 | *Aegithalos concinnus* | 37 |
| *Carduelis sinica* | 14 | *Alcippe morrisonia* | 36 |
| *Cinclus pallasii* | 2 | *Dendrocopos major* | 12 |
| *Emberiza cioides* | 5 | *Garrulax monileger* | 5 |
| *Emberiza rustica* | 7 | *Garrulax pectoralis* | 10 |
| *Emberiza spodocephala* | 3 | *Garrulus glandarius* | 4 |
| *Hemixos castanonotus* | 18 | *Leiothrix lutea* | 8 |
| *Hypsipetes mcclellandii* | 17 | *Monticola solitarius* | 3 |
| *Lonchura striata* | 5 | *Muscicapa sibirica* | 2 |
| *Parus major* | 8 | *Myophonus caeruleus* | 2 |
| *Parus spilonotus* | 3 | *Paradoxornis gularis* | 20 |
| *Parus venustulus* | 3 | *Paradoxornis webbianus* | 23 |
| *Passer rutilans* | 20 | *Picus canus* | 12 |
| *Phasianus colchicus* | 5 | *Pomatorhinus ruficollis* | 3 |
| *Phoenicurus auroreus* | 12 | *Stachyris ruficeps* | 10 |
| *Rhyacornis fuliginosus* | 8 | *Turdus hortulorum* | 32 |
| *Spizixos semitorques* | 23 | *Yuhina castaniceps* | 12 |
| *Tarsiger cyanurus* | 5 | *Zoothera dauma* | 17 |
| *Urocissa erythrorhyncha* | 22 |  |  |
